# Acupuncture for adolescents with mild-to-moderate myopia: study protocol for a randomized controlled trial

**DOI:** 10.1186/1745-6215-15-477

**Published:** 2014-12-05

**Authors:** Yan Wang, Yun-xian Gao, Qi Sun, Qian Bu, Jing Shi, Ya-ni Zhang, Qin Xu, Yan Ji, Min Tong, Guang-li Jiang

**Affiliations:** Xinjiang Academy of Traditional Chinese Medicine, No.116, Yellow River Road, Shayibake District, Ürümqi, 830000 China; Medical Research Design and Data Analysis Center of Traditional Chinese Medicine Hospital, Affiliated to Xinjiang Medical University, No.116, Yellow River Road, Shayibake District, Ürümqi, 830000 China; The Medical Ethics Committee of Traditional Chinese Medicine Hospital, Affiliated to Xinjiang Medical University, No.116, Yellow River Road, Shayibake District, Ürümqi, 830000 China

**Keywords:** Adolescent, Low-to-moderate myopia, Acupuncture, Randomized controlled trial

## Abstract

**Background:**

Myopia is a public health problem worldwide and its incidence increases with age. The use of acupuncture to treat myopia is a common practice in China, however, the use of acupuncture to treat myopia is disputed in other parts of the world. This study aims to determine the safety of acupuncture to treat myopia and its efficacy over six months.

**Methods/Design:**

A randomized, parallel, single-center, assessor- and statistician-blinded, controlled clinical trial will be performed. A total of 100 teenagers, between seven and 12 years of age, with mild-to-moderate myopia and spherical lenses <-6.00 D and cylindrical lenses <-1.50 D will be selected from the Xinjiang Uygur Autonomous Region Institute of Traditional Chinese Medicine, a grade III level A teaching hospital in Urumqi, Xinjiang, China (Xinjiang Medical University Affiliated Hospital of Traditional Medicine). The subjects will be randomly assigned to two different groups (control and acupuncture groups), each group containing 50 subjects. The subjects in both groups wear single-vision corrective lenses. In the acupuncture group, acupuncture will be performed daily for nine consecutive days on five points (bilateral Cuanzhu, Tongziliao, Sibai, Muchuang, and Hegu), followed by no treatment for one day. Six cycles of treatment will be undertaken continuously for a total of 60 days. Following 60 days of treatment, a follow-up period of six months will be included. The primary outcome will be diopter determination. The secondary outcomes will include distance visual acuity, axial length, lens thickness, ciliary body thickness, and subjective symptoms of the eyes and entire body. The main time points for the evaluation of clinical efficacy will be the first, third, and sixth months after treatment.

**Discussion:**

This study will provide clinical observations of various indices following the use of acupuncture to treat adolescents with mild-to-moderate myopia, as well as information on the safety of acupuncture.

**Trial registration:**

Chinese Clinical Trial Registry (identifier: ChiCTR-TRC-13003448; registration date: 7 August 2013).

## Background

Myopia is becoming increasingly common and has become a worldwide public health issue [1]. In the United States and Europe, the incidence of myopia is 20% to 50% [2]; in Asian countries, such as mainland China, Taiwan, Hong Kong, and Singapore, the incidence of myopia is 60 to 80% [3–5]. Over the past 50 to 60 years, the incidence of myopia in East and Southeast Asian countries has rapidly increased. Approximately 80 to 90% of urban adolescents in these countries have myopia on graduating from high school [1]. Myopia often occurs between six and eight years of age, develops rapidly between 13 and 16 years of age, and stabilizes after 16 years of age [6]. Treatment of myopia in modern medicine is mainly optometric, and includes the use of spectacles (single-vision lenses (SVLs) and progressive addition lenses [7] and contact lenses [8, 9]. Refractive surgeries include laser-assisted *in situ* keratectomy, photorefractive keratectomy [10–12], and posterior chamber phakic intraocular lenses [13]. However, while the most commonly used treatment method (optometric) can increase the best corrected visual acuity, optometric methods are unable to improve the uncorrected visual acuity. Surgery can help eliminate the need for glasses, but may lead to complications, as indicated by long-term follow-up studies [13, 14]. There is also an age limit for performing such surgery, and it is considered as the last treatment choice for teenagers.

Acupuncture is highly valued in traditional Chinese medicine, and enjoys a favorable history of more than 2,500 years. Acupuncture has helped millions of patients around the world, and has been shown to be effective in clinical treatment. In 1980, the World Health Organization recommended acupuncture as an effective alternative therapy [15]. Previous studies have confirmed that acupuncture, ear pins, or ear massage can help treat mild myopia (<-3.0 D) [16]. There are published reports on the use of acupuncture in the treatment of adolescent myopia, but most studies use visual acuity as the primary outcome measure. Indeed, visual acuity, as determined by eye examination, is easily influenced by many external factors. Therefore, we have designed a randomized controlled trial based on the physiology and pathology of myopia. In addition to using distance visual acuity, diopter, and axial length (AL) [17] as indices of clinical observation, indices such as ciliary body thickness [18] and lens thickness [19] will also be utilized to obtain more objective and quantitative results. This trial aims to confirm the effectiveness and safety of acupuncture in the treatment of adolescent myopia.

## Methods/Design

### Ethics and dissemination

This trial was designed and will be implemented in accordance with the relevant provision on the protection of rights and interests of the subjects in the Declaration of Helsinki version 08 and the ‘*Biological Ethics Review Method Involving Humans*’ by the Ministry of Health of the People's Republic of China (2007). The implementation of this project has been reviewed and approved by the Ethics Review Committee of Xinjiang Medical University Affiliated Hospital of Traditional Chinese Medicine. The Certificate number of the ethics review is 2013 XE008-1. Written informed consent will be obtained from the guardians of each of the participants.

### Study design

**Figure 1:**
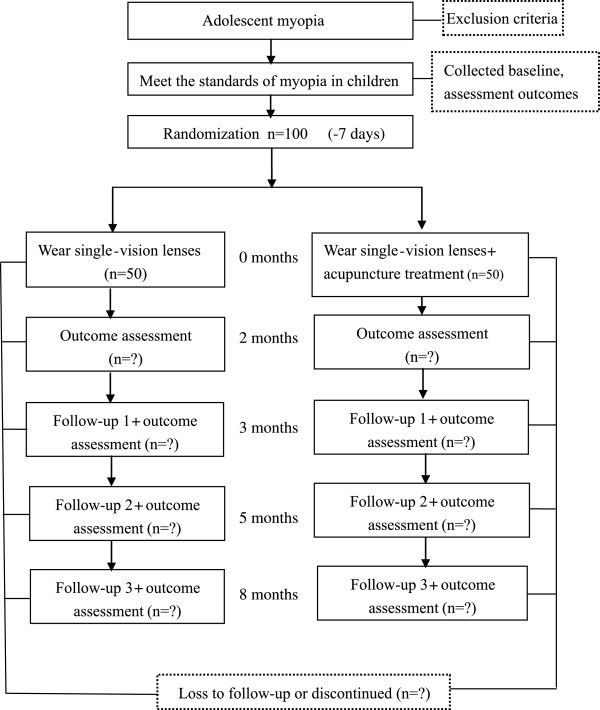
**Flow of participants.**

This study will be a randomized, parallel, single-center, assessor- and statistician-blinded, controlled trial. The included subjects will be assigned to groups in a 1:1 ratio. The subjects (n = 100) will be randomly assigned to experimental (n = 50) and control groups (n = 50). The experiments began in January 2013 and will be completed in December 2014. The aim of this study is to objectively evaluate the clinical efficacy of acupuncture at five acupuncture points (bilateral Cuanzhu, Tongziliao, Sibai, Muchuang, and Hegu) in adolescent teenagers with mild-to-moderate myopia (Table 1, Figure 1). This report will be compiled according to the SPIRIT (Standard Protocol Items: Recommendations for Interventional Trials) 2013 Statement ‘*Defining Standard Protocol Items for Clinical Trials*’ [20].

**Table 1:** **Time of visit and data collection**

Data obtained/recorded	Baseline	Treatment phase	Visit 1	Visit 2	Visit 3
	-7 days	1-2 months	3 months	5 months	8 months
Signed informed consent	×				
Medical history	×				
Met the diagnostic standard/exclusion criteria	×				
General record	×				
Diagnosis	×				
Visual acuity	×		×	×	×
Diopter	×		×	×	×
Axial length	×		×	×	×
Lens thickness	×		×	×	×
Ciliary body thickness	×		×	×	×
Skin damage	×	×	×	×	×
Eye symptoms and systemic symptoms	×	×	×	×	×
Randomization	×				
Group therapy	×				
Case report form	×		×	×	×
Adverse event		×	×	×	×
Close-out					×

### Recruitment

This study will be conducted in the Department of Ophthalmology, Xinjiang Uygur Autonomous Region Institute of Traditional Chinese Medicine (Xinjiang Medical University Affiliated Hospital of Traditional Chinese Medicine, a grade III level A teaching hospital). Adolescents with mild-to-moderate myopia will be recruited from the 27th Elementary School near the Xinjiang Uygur Autonomous Region Institute of Traditional Chinese Medicine after an ophthalmologic examination. Inclusion criteria will be as follows: the result of retinoscopy is myopic refractive error; distance visual acuity can be corrected to normal using negative spherical or cylindrical lenses; near visual acuity and other visual functions must be normal; myopia diopter spherical lenses must be between -0.5 D and -6.00 D, and cylindrical lenses <1.5 D, as measured under cycloplegia; and adolescents must be between seven and 12 years of age. Exclusion criteria will be as follows: subjects with serious systemic diseases, such as cerebrovascular, liver, kidney, hematopoietic system, and psychiatric diseases; long-term use of other related drugs or treatments, which have not been terminated; diopter < -0.5 D, or combined with pathologic myopia-related fundus changes and/or significant visual function impairment; and the affected eyes have other diseases which affect the determination of acupuncture efficacy.

### Withdrawal conditions

The withdrawal conditions will be as follows: the subject has an allergic or serious adverse reaction and, based on the judgment of the physician, the trial should be terminated; if deterioration occurs during the trial and, based on the judgment of the physician, the trial should be terminated; or the guardians of the subject change medicine at their own will or use Chinese or Western drugs prohibited by this trial. Subjects can terminate participation in the trial as follows: subjects who decide to terminate participation in the trial for any reason; subjects or their guardians who are not willing to, or cannot continue the clinical trial, and thereby terminate participation in the trial; and subjects, although not required to terminate participation in the trial, no longer accept the treatment and examinations. The case record forms (CRFs) for the withdrawn cases will be preserved. The results of the last trial will be considered to be the final results. The data on the clinical efficacy and adverse reactions of acupuncture will be fully analyzed.

### Standards of excluded and dropout cases

#### Standards of dropout cases

All participants have the right to terminate the clinical trial at any time. Subjects who do not complete the clinical trial will be considered dropout cases. The common causes of dropouts are as follows: adverse events, lack of efficacy, violation of the trial plan, lost to follow-up (including the subjects or their guardians who decided to terminate the treatment), and other reasons.

#### Dropout cases

For dropout cases, the researchers will specify the reason for dropping out in the CRF. At the same time, the researchers will contact the guardians and complete the assessment items; the causes for dropping out will be made clear and specified in the CRF. The observational data on all dropout cases will be preserved for statistical analysis at the end of the trial.

#### Excluded cases

If the cases included conform to one of the following, they will be excluded: mistakenly included; and lack of treatment. The reason for a case being excluded will be specified. The CRF will be preserved for future reference. Statistical analysis of the efficacy data will not be carried out, however, the subjects who receive at least one treatment session will be included in the analysis of adverse reactions.

### Recruitment strategies

The subjects will be informed of the details regarding the trial plan and those who voluntarily participate in the trial will receive free ophthalmologic examinations. Following optometric evaluation, these subjects will receive the highest discount (30% off) on glasses. Subjects in the experimental group will receive a 50% discount on acupuncture treatment. All subjects who receive a follow-up eye exam will not pay a fee and will be permitted to be fast-tracked. The subjects who will be included in the experimental group will receive treatment any time between 10:00 and 19:00 (Beijing time). If adverse events occur during treatment, the treatment will be immediately terminated, and help will be obtained from the emergency department. According to the specific condition which occurs after treatment, a consultation involving at least three experts will be carried out to decide whether or not to continue the clinical trial. Subject compliance will be determined based on whether or not the subject receives due treatment. The researchers will ensure that the subjects in the experimental group fully understand the importance of regular acupuncture and compliance. During the observation period, the use of drugs or other therapies to treat mild-to-moderate myopia will be prohibited.

### Randomization and allocation concealment

#### Randomization

Randomized sequences will be generated by the Medical Research Design and Data Processing Center of Xinjiang Medical University Affiliated Hospital of Traditional Chinese Medicine as the third party. SPSS 19.0 (SPSS Inc., Chicago, Illinois, United States) software (seed = 20,000) will be used to generate serial numbers for the subjects; 100 random numbers and the allocation sequence table of the randomized group will be preserved as blind codes. Subjects will be assigned to the experimental (n = 50) and control groups (n = 50) at a ratio of 1:1.

### Allocation concealment

Based on the randomized sequences, the third party will place paper strips with black characters on a grey background into non-transparent envelopes which are sealed with adhesive.

### Implementation

The randomized sequences will be generated by the third party. The researchers consist of personnel from the Department of Ophthalmology at the Xinjiang Uygur Autonomous Region Institute of Traditional Chinese Medicine. The subjects will be allocated into experimental and control groups by researchers according to the randomized sequences generated. One copy of the blind codes containing the random serial numbers will be preserved by the third party and research director. The physician responsible for determining the primary and secondary outcomes will be blind to the grouping of subjects and will not participate in the acupuncture treatment. All outcomes will be assessed and the results will be analyzed by a statistician blinded with respect to the allocations of the different treatments.

### Intervention

**Figure 2:**
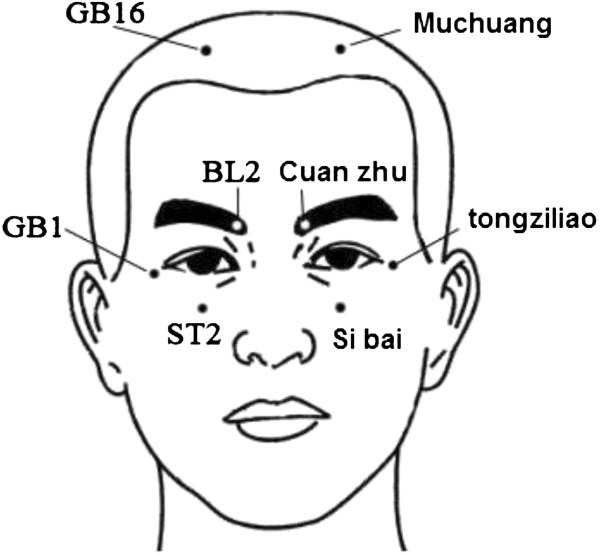
**Location of facial acupoints.** Four points are used for adolescents in the acupuncture group which includes bilateral Cuanzhu (BL2), Tongziliao (GB1), Sibai (ST2), and Muchuang (GB16).

**Figure 3:**
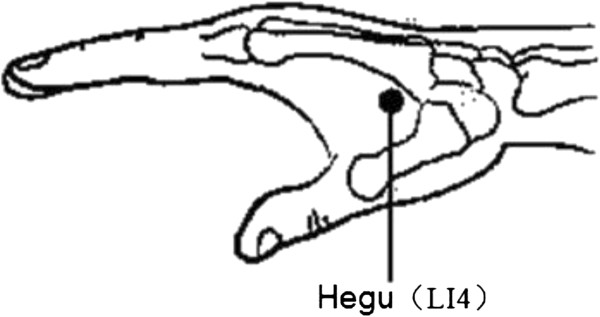
**Location of hand acupoint.** Bilateral Hegu (LI4) is used for adolescents in the acupuncture group.

Subjects in the acupuncture group will wear SVLs. Acupuncture will be performed at five acupuncture points (bilateral Cuanzhu (BL2), Tongziliao (GB1), Sibai (ST2), Muchuang (GB16), and Hegu (LI4), see Figures 2 and 3). Alcohol (75%) will be used to routinely disinfect the local skin at the acupuncture points and 0.25 mm × 0.4 mm disposable sterile acupuncture needles (Suzhou Acupuncture & Moxibustion Appliance Co., Ltd., Suzhou, China) will be used at acupuncture points Sibai and Hegu to a depth of 0.5 to 1.0 cm. After achieving *de-qi* (Subjects have a feeling of soreness, fullness, aching, heaviness at and around the acupoints), the needle will be held in position for 20 minutes. Cuanzhu will be acupunctured to a depth of 0.3 to 0.5 cm. Tongziliao and Muchuang will be acupunctured to a depth of 0.1 to 0.3 cm. After achieving *de-qi*, the needle will be held in position for 20 minutes. Acupuncture will be carried out once a day between 10:00 and 19:00 (Beijing time). One cycle of treatment will last for nine days, followed by one day of no treatment. Six cycles of treatment will be undertaken continuously for a total of 60 days. The acupuncturist in this study, a physician with 18 years of experience in clinical acupuncture, will not participate in objective examinations, such as visual acuity. Subjects in the control group will wear SVLs without any other treatment affecting visual acuity. Observations will be carried out for two consecutive months.

### Data collection

Baseline data will be collected before the first treatment. The information collected will include gender, date of birth, ethnicity, height, birth weight, current body weight, premature or full-term baby, parental myopia, sibling myopia, time of reading at a close distance, time of daily outdoor activities, performance of daily eye exercises, room lighting, and bad reading habits (reading while lying, walking, or riding on the bus).

### Primary outcome

The primary outcome in this trial will be diopter measurement [21]. After mydriasis using cyclopentolate hydrochloride eye drops (15 ml; Alcon-Couvreur, Puurs, Belgium), a comprehensive refractometer (AOS-1,500; Nidek, Aichi, Japan) will be used to conduct objective optometry (computer optometry and retinoscopy), then subjective optometrics. Subjective optometrics will be divided into the following two parts: optometrics will be conducted on a single eye (the right eye followed by the left eye), and a bilateral eye balance examination. Subjective optometrics of a single eye will be divided into the following three stages: the initial maximum plus the maximum visual acuity (MPMVA) will be identified, Jackson cross cylindrical lenses will be used to determine the axial direction and degree of the cylindrical lens, and the MPMVA will be repeated. The results will be recorded after optometrics. When the pupils recover, a re-examination will be carried out (three days after the use of cyclopentolate hydrochloride eye drops). After re-examination, the glasses will be tried again. The degree of the glasses will be marked on the glass trial frame, to be chosen by the subjects based on subjective judgment. Finally, the diopter of the glasses and prescription glasses will be determined. The optometrist will not participate in other parts of the trial. The main time points for evaluation of clinical efficacy will be the first, third, and sixth months after two months of treatment.

### Secondary outcomes

The secondary outcomes in this study will include distance visual acuity [22], AL [17], lens thickness [18], and ciliary body thickness [19]. These indices will be measured before treatment, and at the first, third, and sixth months after two months of treatment. The objective examinations will be recorded in the CRFs. The main time points of evaluation will be the sixth month after treatment or when the trial is terminated.

Distance visual acuity will be measured using a standard logarithmic visual acuity test chart (TR-B; Tongrui Medical Equipment Co., Ltd., Tianjin, China), under sufficient lighting to measure the parameters of visual acuity, with a 5-m distance to the visual acuity test chart. All visual acuity examinations in this study will be carried out by one nurse in a fixed place who will not participate in acupuncture treatment or clinical examinations. The specific details of the subjects will not be disclosed to the nurse.

In order to measure AL and lens thicknesses, the subject will be placed in the supine position. Oxybuprocaine Hydrochloride Eye Drops (20 ml, Santen Pharmaceutical Co. Ltd. Suzhou, China) will be applied to the eye to be examined, and topical anesthesia will be applied. The subjects will be asked to watch the red light at the top of the probe with the eye to be examined. A disinfected ultrasound probe will be placed on the surface of the cornea. Sound waves will pass through the middle of the corneal vertex, the center of the lens, and the vitreous body, to reach the macular fovea. The sound beam will be kept in a vertical orientation. The AL and lens thicknesses will be measured. Ten sets of values will be selected, with a measurement error within 0.1 mm [22]. The lens thickness is the axial distance from the front surface to the posterior surface of the lens [19, 23]. An ophthalmic ultrasound instrument (CINESCAN; Quantal Medical, Paris, France) will be used for these measurements in the proposed study. Ten measurements of a particular index will usually be made, and these 10 measurements will be recorded and the average value obtained. The AL and lens thickness measurements will be carried out by one full-time physician, on a fixed instrument, and at a fixed location. The physician will not participate in visual acuity examinations and acupuncture treatment, and the specific details with respect to the grouping of subjects will not be disclosed to the physician.

In order to measure ciliary body thickness, oxybuprocaine hydrochloride eye drops will be applied to the eye to be examined. After topical anesthesia is applied, a disinfected eye cup of appropriate size will be placed beneath the upper eyelid. At this time, the subjects will be asked to move the eye downward to prevent scratching the cornea. The lower eyelid will then be pulled. The eye cup will be placed beneath the lower eyelid, so that it is immobilized between the eyelids and the eyeball. Distilled water will be poured into the eye cup. The contralateral eye will gaze at the red indicator light on the nasal side. An ultrasound biomicroscope (MD-300 L; Meda Co. Ltd., Tianjin, China) will be used. A 50 mhz transducer with 4 to 5 mm tissue penetration depth will be used to measure ciliary body thickness at the temporal corneoscleral limbus of the eye to be examined. The image will then be obtained. The built-in ruler will be used to measure ciliary body thickness (vertical distance) 2 mm behind the scleral spur [24, 25]. The position of the bilateral temporal side (nine points in the right eye, three points in the left eye) will be measured once manually and the results will be recorded. The same instrument will be used for all measurements. The measurement of ciliary body thickness in this research will be completed by one full-time physician, using a fixed instrument, in a fixed place. The physician will not participate in visual acuity examinations or acupuncture treatment, and the specific details of the subjects will not be disclosed to the physician.

### Data management

An electronic CRF will be designed on the Research Manager (Clinical Trial Management Public Platform, Chengdu, China) clinical trials public management platform. A printed version will also be obtained. According to data management provisions, the electronic data will be saved for at least 30 years in the form of a CD.

The electronic CRF will be designed on the Research Manager clinical trials public management platform. The examination, input, and modification of case observation tables will be registered in a timely registered to ensure that the case observation tables are returned in a timely fashion and entered into the database. The original documents will be stored in the Department of Ophthalmology. The data administrator will be in charge of data entry. To guarantee the accuracy of the data, two data administrators will enter and check the data independently. In the case of questions arising from the CRFs, the data administrator will complete a data recheck query (DRQ) and issue an enquiry to the researchers through the clinical supervisor. The researchers will answer and return the DRQ as soon as possible. The data administrators will then revise, confirm, and enter the data according to the researcher’s reply and a DRQ will be issued again if necessary. All scientific research documents will be stored in special-purpose filing cabinets and locked to guarantee the security of the files stored. Custody of the files will be maintained through an on-the-spot inspection method. Personnel will be specifically assigned to manage the files. Various written materials will be saved according to needs. The information will be stored in a safe and orderly means to avoid water damage, staining, and crimping. The files will be kept clean and in good condition without damage. After the data is entered, lost or unreliable data will be subject to inquiry. Patients with multiple records will be inspected. The data will be validated by error analysis. Data management software will be used to build the database. Personnel will be specifically assigned to oversee the management of electronic data and organize the clinical research team in terms of data entry, verification, reporting, and answering questions.

### Harms

In the proposed study, adverse events, included bleeding, hematoma formation, fainting, severe pain, and local infection, will be reported [26]. If adverse events occur during the observation period, details of these adverse effects will be recorded in the CRFs.

### Sample size and statistical analysis

#### Sample size

Based on the myopia diopter described in the literature [27], the sample size will be estimated using the formula for superiority test sample size for two quantitative samples [28]. The inspection significance level α = 0.05 (two-sided) with a combined standard deviation (S) of 0.25, will be obtained according to the literature, where *u*_1*-α*_, *u*_1-β_ are the bounds of the one-sided standard normal deviation, *u*_1–0.05_ = 1.645, and *u*_1-o.20_ = 0.845. *δ*_u_ is the effective boundary (*δ*_u_ >0), assuming *δ*_u_ = 0.2; θ is the estimate of the overall mean difference value of the acupuncture and control groups, and will be set at 3.0 according to the literature [27]. By substitution into the above formula with n = 79 and an expected dropout rate of 20%, a total of 100 cases will be included. The sample ratio between the groups will be 1:1.

### Statistical analysis

All statistical analyses will be performed using SPSS 17.0 statistics software. For quantitative data, the distribution pattern and homogeneity of variance will be examined. If there is a symmetric distribution, the mean (M) ± standard deviation will be used for statistical description, otherwise the M interquartile range (IQR) will be used to describe the trend of concentration and amount of scatter. If this condition is met, the Student's t-test will be used to compare the differences between the quantitative indices between the groups, otherwise the Wilcoxon rank-sum test will be used. For categorical data (binary classification and multiple classification), the composition ratio will be used for statistical description. Based on the observation of a theoretical frequency distribution, the χ^2^ or Fisher's exact test will be used, depending on the specific situation. The ranked data will be analyzed using the Wilcoxon rank-sum test.

Primary and secondary outcomes will be analyzed by the superiority test in accordance with the principle of intention-to-treat, using Per Protocol Set (PPS) and Full Analysis Set (FAS) datasets. The indices of safety evaluation will be analyzed using the Save Set (SS) dataset. The significance level in this study will be set at α = 0.05, and the one-sided test will be used. The detailed statistical analytical methods for primary and secondary outcomes and baseline data are shown in Table 2 (variables, measures, and methods of analysis).

**Table 2:** **Variables, measures, and methods of analysis**

Variable/outcome	Hypothesis	Outcome measure	Methods of analysis
1) Primary	Intervention improved outcome from baseline to 7 days	Adherence% (>80%) [binary]	
Diopter	Diopter reduced or increased after treatment	Diopter [continuous]	t-test (rank-sum test) or repeated measurement
2) Secondary	Intervention improved outcome from baseline to 7 days	Adherence% (>80%) [binary]	
Visual acuity	Visual acuity did not increase or decrease after treatment	Visual acuity changes [continuous]	t-test (rank-sum test) or repeated measurement
Axial length	Axial length was not increased	Axial length changes [continuous]	t-test (rank-sum test) or repeated measurement
Lens thickness	Lens thickness was not increased	Lens thickness changes [continuous]	t-test (rank-sum test) or repeated measurement
Ciliary body thickness	Ciliary body thickness was not increased	Ciliary body thickness changes [continuous]	t-test (rank-sum test) or repeated measurement
Eye symptoms and systemic symptoms	No adverse symptoms	No adverse symptoms	Chi-squared test or Fisher’s exact test
Compliance	Compliance	Cure rate [binary]	Chi-squared test or Fisher’s exact test
Combination therapy/treatment	Improvement occurred	Whether other drugs were used during treatment [binary]	Chi-squared test or Fisher’s exact test
Adverse event	Improvement occurred	Presence of drug-related adverse event [binary]	Chi-squared test or Fisher’s exact test
3) Baseline analyses:		Adherence% (>80%) [continuous]	
Age		CRF [continuous]	t-test (rank-sum test)
Weight and height		CRF [continuous]	t-test (rank-sum test)
Female versus male		CRF [binary]	Chi-squared test or Fisher’s exact test
Race		CRF [more categories]	Chi-squared test or rank-sum test
Birth weight		CRF [continuous]	t-test (rank-sum test)
Preterm children		CRF [binary]	Chi-squared test or Fisher’s exact test
Parental myopia or not		CRF [binary]	Chi-squared test or Fisher’s exact test
Sibling myopia or not		CRF [binary]	Chi-squared test or Fisher’s exact test
Daily close reading time		CRF [continuous]	t-test (rank-sum test)
Outdoor time		CRF [continuous]	t-test (rank-sum test)
Eye exercises every day or not		CRF [binary]	Chi-squared test or Fisher’s exact test
Daily sleep time		CRF [continuous]	t-test (rank-sum test)
Room light conditions		CRF [binary]	Chi-squared test or Fisher’s exact test
Poor reading habits		CRF [more categories]	Chi-squared test or rank-sum test

CRF, case report form ; rank-sum test, Wilcoxon rank-sum test; t-test, Student’s t-test.

### Monitoring

In the proposed study, a Data Monitoring Committee (DMC) will be installed; the quality control office of the entire project will be set up in DMC. A quality control team will also be set up and a person-in-charge will be appointed (members are the researchers, consisting of personnel from the Ethics Committee, statistical analysis personnel, and clinical professionals (five people in total)) to identify problems in the project implementation process in a timely manner and to implement the corresponding countermeasures. The collected data will be examined, and the researchers will be supervised to control bias.

### Protocol amendments and confidentiality

Notification of major modifications to the project (inclusion criteria, outcomes, and analytical method) will be provided to the Ethics Committee, subjects, register, and publisher. The researchers promise to keep the information and technology involved in the files confidential. The personal information on the subjects will be strictly confidential, and not disclosed under any circumstances. Moreover, a confidentiality agreement will be signed.

### Declaration of interest

This research will be funded by the Youth Science and Technology Talents Project of Xinjiang Uygur Autonomous Region Health Department. The Xinjiang Uygur Autonomous Region Health Department and the researchers who take part in the proposed study will not have any conflicts of interests. The researchers will not have any conflicts of interests with the subjects and their guardians.

### Ancillary and post-trial care

When emergencies or adverse reactions are observed, the researchers will carry out the corresponding countermeasures according to the symptoms to ensure the safety of the subjects. The results of the treatment administered will be reviewed by the project director. The treatment and the results of treatment will be recorded in detail in the CRFs, which will later be signed. If adverse reactions related to treatment occur and need treatment, the research team will meet the costs of treatment according to relevant regulations. Patients who experience adverse effects are given economic compensation.

## Discussion

Traditional Chinese medicine theory states that the ‘meridian is the channel through which *qi* and blood circulate. An acupoint is the site where the *qi* of the viscera is transferred and concentrated’ [29, 30]; the meridian is the pathway for the body surface to connect and interact with the internal organs; and the acupuncture point is a specific point on the meridians, forming a special functional relationship with the corresponding viscera and the related surface structure through the meridians. Stimulation of the acupuncture point can have a specific treatment effect. Therefore, traditional Chinese medicine purports that ‘the meridian circulation is closely related to the amount of meridian *qi* concentrated’ [31]. Acupuncture treatment has a clinical effect, and acts on specific meridians and collaterals and specific acupuncture points [31]. Some studies suggested that acupuncture at visual-related acupoints could modulate optical physiology [32]. Acupuncture points are specific, but in a relative sense [30]. Therefore, in the proposed study the local points (Cuanzhu, Tongziliao, Sibai, Muchuang, and Hegu) will be acupunctured simultaneously, and the clinical efficacy of acupuncture treatment in adolescent mild-to-moderate myopia will be determined.

The advantages of the proposed study will be four-fold. Firstly, Cuanzhu, Tongziliao, Sibai, Muchuang, and Hegu will be acupunctured to study the specific efficacy and safety of acupuncture in adolescent mild-to-moderate myopia. Secondly, the acupuncturist involved in the proposed study will have more than 10 years of experience in clinical acupuncture. The acupuncture procedure in all subjects in the experimental group will be carried out by the acupuncturist, which will ensure consistency of acupuncture treatment. Thirdly, the subjects will mainly be children from a primary school near the hospital, which will help to ensure regular follow-up visits and examinations of the subjects. Fourthly, previous clinical experience has shown that acupuncture treatment of adolescent simple myopia has almost no side effects.

The limitations of the proposed study are three-fold. Firstly, due to a limited budget, the proposed study will not be a multicenter trial, which will influence the external validity of the conclusions. Secondly, common fake acupuncture methods include neighboring sham acupuncture, non-specific site acupuncture, superficial acupuncture, minimal acupuncture, and a placebo needle; all of these methods could be used as a control, but with obvious deficiencies [33]. Therefore, in the proposed study a sham acupuncture group will not be included. Quantitative, objective indices, such as diopter, AL, lens thickness, and ciliary body thickness will be selected as the outcomes to evaluate the clinical efficacy at a reduced bias. Thirdly, in the proposed study, 100 cases will be included and followed up on for six months. To prevent bias caused by loss-to-follow-up, the following measures will be adopted: first, the subjects will be from an elementary school near the hospital. Second, before the subjects are entered into the proposed study, their guardians will fully understood the specific requirements of the follow-up schedule, as outlined in the informed consent form. Once the subjects are included in the study, communication with the guardians will be strengthened by phone calls or email. The subjects will be informed of the follow-up examination time and place in advance. The subjects will receive the follow-up examination immediately after arriving.

This will be a randomized, parallel controlled trial, and it is anticipated that acupuncture treatment of adolescent mild-to-moderate myopia will be shown to be safe and effective. The proposed study will demonstrate the specificity of the meridians and acupuncture points and provide evidence-based medical evidence on the use of acupuncture to treat myopia.

## Trial status

The first participant was included on 27 November 2013 and to date, six participants have been recruited. The study will be finished on 30 June 2015.

## Abbreviations

AL: Axial length
CRF: Case record form
DRQ: Data recheck query
MPMVA: Maximum plus to maximum visual acuity
SVLs: Single-vision lenses.
